# Screening patients requiring secondary lumbar surgery for degenerative lumbar spine diseases: a nationwide sample cohort study

**DOI:** 10.1038/s41598-024-51861-7

**Published:** 2024-01-14

**Authors:** Hangeul Park, Juhee Lee, Yunhee Choi, Jun-Hoe Kim, Sum Kim, Young-Rak Kim, Chang-Hyun Lee, Sung Bae Park, Kyoung-Tae Kim, John M. Rhee, Chi Heon Kim

**Affiliations:** 1 Department of Neurosurgery, Seoul National University Hospital, 101, Daehak-ro, Jongno-gu, Seoul, 03080 Republic of Korea; 2 Division of Medical Statistics, Medical Research Collaborating Center, Seoul National University Hospital, 101, Daehak-ro, Jongno-gu, Seoul, 03080 Republic of Korea; 3 Department of Neurosurgery, Seoul National University College of Medicine, 103, Daehak-ro, Jongno-gu, Seoul, 03080 Republic of Korea; 4 Department of Neurosurgery, Seoul National University Boramae Hospital, Boramae Medical Center, 20, Boramae-ro 5-gil, Dongjak-gu, Seoul, 07061 Republic of Korea; 5 Department of Neurosurgery, School of Medicine, Kyungpook National University, Kyungpook National University Hospital, Daegu, Republic of Korea; 6 Department of Orthopaedic Surgery, Emory University School of Medicine, Atlanta, GA 30322 USA; 7 Department of Medical Device Development, Seoul National University College of Medicine, 103 Daehak-ro, Jongno-gu, Seoul, 03080 Republic of Korea

**Keywords:** Health policy, Chronic pain, Neurological disorders, Health care economics

## Abstract

This study aims to identify healthcare costs indicators predicting secondary surgery for degenerative lumbar spine disease (DLSD), which significantly impacts healthcare budgets. Analyzing data from the National Health Insurance Service-National Sample Cohort (NHIS-NSC) database of Republic of Korea (ROK), the study included 3881 patients who had surgery for lumbar disc herniation (LDH), lumbar spinal stenosis without spondylolisthesis (LSS without SPL), lumbar spinal stenosis with spondylolisthesis (LSS with SPL), and spondylolysis (SP) from 2006 to 2008. Patients were categorized into two groups: those undergoing secondary surgery (S-group) and those not (NS-group). Surgical and interim costs were compared, with S-group having higher secondary surgery costs ($1829.59 vs $1618.40 in NS-group, P = 0.002) and higher interim costs ($30.03; 1.86% of initial surgery costs vs $16.09; 0.99% of initial surgery costs in NS-group, P < 0.0001). The same trend was observed in LDH, LSS without SPL, and LSS with SPL (P < 0.0001). Monitoring interim costs trends post-initial surgery can effectively identify patients requiring secondary surgery.

## Introduction

Degenerative lumbar spine disease (DLSD) is one of the most common musculoskeletal conditions that affect the lower back and is characterized by the progressive deterioration of intervertebral discs, facet joints, and other structures in the lumbar region^1,2^. This degenerative process can cause a variety of symptoms, including back pain, leg pain, tingling, and weakness, which can significantly impact an individual's quality of life^3,4^. While various treatment options exist for DLSD, including conservative management such as physical therapy and medication, some patients may require surgical intervention to alleviate their symptoms and improve their quality of life^5,6^. Surgical procedures such as lumbar fusion or discectomy are often effective in providing relief, restoring spinal stability, and improving functional outcomes^7–10^. However, despite the success of initial surgical interventions, a subset of patients may experience recurrent symptoms or the progression of their condition over time. This may necessitate a second surgical procedure, commonly referred to as secondary surgery^11–13^. The need for secondary surgery in DLSD can arise due to various reasons, including adjacent segment disease (ASD), implant failure, persistent or recurrent symptoms, or disease progression^14–17^. Secondary surgeries often require more complex surgical techniques compared to the initial surgery, which can contribute to an increase in healthcare costs including surgical fees, hospitalization costs, and post-operative care^18–21^. The increasing incidence of secondary surgery has raised concerns regarding its impact on healthcare costs^22^. Therefore, screening secondary surgery for DLSD is essential not only for the efficient allocation of healthcare resources and rational medical expenditure but also for formulating appropriate policies regarding the medical costs associated with DLSD. Factors associated with secondary surgery are complex, but there has been no indicator showing the possibility of secondary surgery from the perspective of health insurance. The objective of this study is to propose indicators for screening patients requiring secondary surgery for DLSD, focusing on the aspect of increased healthcare costs, using data from the National Health Insurance Service-National Sample Cohort (NHIS-NSC) of the Republic of Korea (ROK).

## Methods

### Data source

The data for this study were derived from the National Health Insurance Database (NHID), which records personal information, demographics, and medical treatment data for all Korean citizens. In the ROK, all citizens have been beneficiaries of the NHIS for more than 20 years, and the NHIS covers both Western and Oriental medicine^23–25^. Because the NHIS follows a fee-for-service payment system, all nationwide inpatient and outpatient data on diseases and services (i.e., procedures and surgeries) are coded and registered in the National Health Insurance Corporation (NHIC) database and the Health Insurance Review & Assessment Service (HIRA) database^23–28^. The disease codes in the database adhere to the 10th version of the International Classification of Diseases (ICD-10), and procedure codes are standardized for billing purposes. Nearly all hospitals providing Western medicine and clinics providing Oriental medicine must follow the guidelines to obtain reimbursement. The detailed surgical and nonsurgical management were determined by the attending physicians^23,25,29^. By using the database, the NHIS-NSC was identified in 2017 for analysis while maintaining representativeness and protecting personal information^28^. The NHIS-NSC represents a representative sample cohort, consisting of 1,000,000 individuals (approximately 2.1% of the total Korean population) randomly selected from a population of 48,438,292 in 2006 (https://nhiss.nhis.or.kr/bd/ab/bdaba021eng.do)^28^. Systematic stratified random sampling with proportional allocation within each stratum, including sex, age, location, and health insurance, was employed. To ensure privacy, the resident registration numbers were replaced with unique eight-digit personal IDs, enabling longitudinal follow-ups for all individuals until 2015. The cohort was updated annually during the follow-up period, and the size of the cohort was maintained. The records for each person in the NHIS-NSC can be traced back to 2002.

### Study population

**Figure 1:**
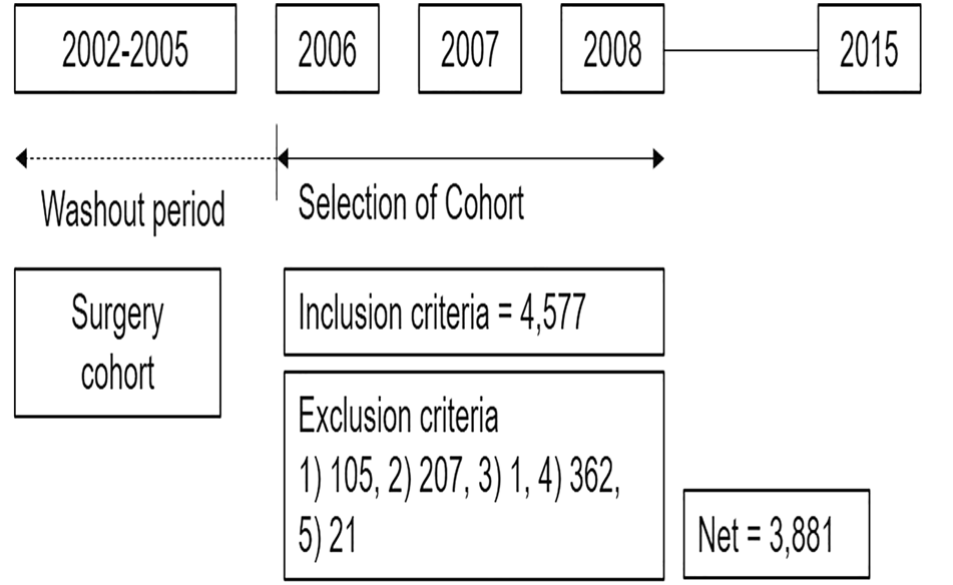
Flow diagram of patients. A total of 4577 patients who underwent surgery for lumbar disc herniation (LDH), lumbar spinal stenosis without spondylolisthesis (LSS without SPL), lumbar spinal stenosis with spondylolisthesis (LSS with SPL), and spondylolysis (SP) between 2006 and 2008 were registered in the surgery cohort. Among the registered patients, the following conditions resulted in exclusions: (1) patients with a history of spinal surgery within the past 3 years (n = 105), (2) patients who had utilized medical services for spinal fracture, pathological fracture, spinal infection, malignancy, or inflammatory joint disease within the past 1 year (n = 207), (3) patients with concomitant rare diseases such as metabolic diseases, blood diseases, or congenital anomalies (n = 1), (4) patients admitted via the emergency room (n = 362), and (5) patients below 18 years of age (n = 21). Finally, the surgical cohort consisted of 3881 patients and was followed up for at least 7 years.

For this study, we utilized a cohort study design established in a previous study^30^. The study included patients diagnosed with lumbar disc herniation (LDH), lumbar spinal stenosis without spondylolisthesis (LSS without SPL), lumbar spinal stenosis with spondylolisthesis (LSS with SPL), and spondylolysis (SP)^30,31^. The disease codes for each diagnosis were as follows: (1) LDH, M51, M472; (2) LSS without SPL, M4800, M4805-8; (3) LSS with SPL, M431, M4315-9; (4) SP, M430, M4306-9. The selection of the surgical treatment cohort involved identifying patients who underwent specific surgical procedures between 2006 and 2008. The codes for each surgical procedure were as follows: (1) open discectomy, N1493; (2) laminectomy, N4199, N2499; (3) endoscopic lumbar discectomy, N1494; (4) spinal fusion, N0466, N1466, N0469, N2470, N1460, and N1469. A total of 4577 patients were selected in the surgical treatment cohort. Among them, patients with the following conditions were excluded: (1) patients with a history of spinal surgery within the past 3 years (n = 105), (2) patients who had utilized medical services with disease indicating spinal fracture, pathological fracture, spinal infection, malignancy, or inflammatory joint disease within the past 1 year (n = 207), (3) patients with concomitant rare diseases such as metabolic diseases, blood diseases, or congenital anomalies (n = 1), (4) patients admitted via the emergency room (n = 362), and (5) patients below 18 years of age (n = 21)^30^. After applying exclusion criteria, 3,881 patients remained in the surgical treatment cohort. After surgery, patients visited clinic for follow-up and may receive additional interventions, physiotherapy or medications depending on their specific needs following the surgery. All patients were followed up for at least 7 years. The patient flow diagram is presented in Fig. 1. Secondary surgery was defined as any kind of lumbar spinal surgery at any lumbar level being performed after initial surgery. However, since the exact lumbar level was not recorded in the registry, treatment failure after initial surgery could include both the index level and the other lumbar levels^24,25,27,29,30,32^. This study was conducted in accordance with the Declaration of Helsinki and the Guideline for Good Clinical Practice. The study protocol was approved by the Seoul National University Hospital ethics committee/institutional review board (2010-076-1164). The Seoul National University Hospital ethics committee/institutional review board approved the exemption of informed consent due to the retrospective nature of this study.

### Statistics

We analyzed direct medical costs for Western and Oriental medicine in two groups: those who had secondary surgery (S-group) and those who did not (NS-group). Costs only considered medical expenses and did not account for societal costs. Initial costs for surgical treatment were incurred during hospitalization for surgery. In the S-group, interim costs covered expenses between initial and secondary surgeries, including consultation fees, procedures, physiotherapy, and medications. In the NS-group, interim costs included expenses after the initial surgery. Costs related to the secondary surgery were specific to the secondary surgery purpose. We compared costs between groups using the Mann–Whitney U test. To find the optimal cutoff for interim costs predicting secondary surgery, we selected the value maximizing sensitivity and specificity based on Youden's index. Statistical analysis was done using SAS version 9.4, with significance set at P < 0.05.

## Results

### Baseline characteristics of the cohort

The characteristics of patients are described in Table 1. The most common disease was LDH (47.85%) followed by LSS without SPL (36.12%), LSS with SPL (13.63%), and SP (2.4%). Open discectomy was the most common surgical technique in all diseases. The initial surgical methods for each diagnosis are shown in Table 2. Fusion surgery was performed in 3.82%, 12.91%, 37.24%, and 43.01% of patients with LDH, LSS without SPL, LSS with SPL, and SP, respectively. The distribution of secondary surgery methods for each diagnosis is presented in Table 3. Secondary surgery was performed in 14.81%, 15.62%, 11.34%, and 6.45% of patients with LDH, LSS without SPL, LSS with SPL, and SP, respectively. Open discectomy was the most common secondary surgical method, and the fusion surgery was more frequently performed than initial surgery in LDH and LSS without SPL; 9.09% (vs 3.82%) and 17.35% (vs 12.91%), respectively.

**Table 1:** The characteristics of the patients.

Category	Number (%)
Age, mean ± SD (range)	53.48 ± 14.45 (18–86)
18–29	287 (7.4)
30–39	445 (11.47)
40–49	715 (18.42)
50–59	862 (22.21)
60–69	1086 (27.98)
70–	486 (12.52)
Sex	
Male	1877 (48.36)
Female	2004 (51.64)
Diagnosis	
Lumbar disc herniation	1857 (47.85)
Lumbar spinal stenosis without spondylolisthesis	1402 (36.12)
Lumbar spinal stenosis with spondylolisthesis	529 (13.63)
Spondylolysis	93 (2.4)
Healthcare facility	
Hospital	2214 (57.05)
General hospital	959 (24.71)
Tertiary referral hospital	520 (13.4)
Clinics	188 (4.84)
Charlson comorbidity index, median (range)	0 (0–14)
Surgical method	
Spondylectomy	5 (0.13)
Anterior fusion	156 (4.02)
Posterior fusion	332 (8.55)
Open discectomy	2571 (66.25)
Laminectomy	636 (16.39)
Endoscopic discectomy	181 (4.66)
Comorbidity	
Parkinson disease	17 (0.44)
Osteoporosis without fracture	1047 (26.98)
Diabetes mellitus	609 (15.69)

**Table 2:** Distribution of initial surgical methods for each diagnosis.

	Spondylectomy	Anterior fusion	Posterior fusion	Open discectomy	Laminectomy	Endoscopic discectomy	Total (%)
Lumbar disc herniation, n (%)	0 (0)	25 (1.35)	46 (2.48)	1535 (82.66)	78 (4.20)	173 (9.32)	1857 (47.85)
Lumbar spinal stenosis without spondylolisthesis, n (%)	2 (0.14)	66 (4.71)	115 (8.20)	743 (53.00)	469 (33.45)	7 (0.50)	1402 (36.12)
Lumbar spinal stenosis with spondylolisthesis, n (%)	2 (0.38)	52 (9.83)	145 (27.41)	252 (47.64)	77 (14.56)	1 (0.19)	529 (13.63)
Spondylolysis, n (%)	0 (0)	14 (15.05)	26 (27.96)	41 (44.09)	12 (12.90)	0 (0)	93 (2.40)
Total (%)	4 (0.10)	157 (4.05)	332 (8.55)	2571 (66.25)	636 (16.39)	181(4.66)	3881 (100)

**Table 3:** Distribution of secondary surgery methods by initial surgery methods depending on each diagnosis.

Diagnosis	Initial surgery methods	Secondary surgery methods						Total
		Spondylectomy	Anterior fusion	Posterior fusion	Open discectomy	Laminectomy	Endoscopic discectomy	
Lumbar disc herniation	Fusion	1 (0.13)	0 (0)	5 (62.5)	2 (25)	0 (0)	0 (0)	8 (2.91)
	Open discectomy	0 (0)	0 (0)	17 (7.39)	184 (80)	21 (9.13)	8 (3.48)	230 (83.64)
	Laminectomy	0 (0)	0 (0)	2 (20)	6 (60)	2 (20)	0 (0)	10 (3.64)
	Endoscopic discectomy	0 (0)	0 (0)	1 (3.7)	22 (81.48)	1 (3.7)	3 (11.11)	27 (9.82)
	Total	1 (0.36)	0 (0)	25 (9.09)	214 (77.82)	24 (8.73)	11 (4)	275 (14.81^†^)
Lumbar spinal stenosis without spondylolisthesis	Anterior fusion	0 (0)	0 (0)	3 (27.27)	6 (54.55)	2 (18.18)	0 (0)	11 (5.02)
	Posterior fusion	0 (0)	0 (0)	5 (35.71)	8 (57.14)	1 (7.14)	0 (0)	14 (6.39)
	Open discectomy	1 (0.79)	0 (0)	16 (12.60)	83 (65.35)	23 (18.11)	4 (3.15)	127 (57.99)
	Laminectomy	1 (1.52)	3 (4.55)	11 (16.67)	28 (42.42)	22 (33.33)	1 (1.52)	66 (30.14)
	Endoscopic discectomy	0 (0)	0 (0)	0 (0)	1 (100)	0 (0)	0 (0)	1 (0.46)
	Total	2 (0.91)	3 (1.37)	35 (15.98)	126 (57.53)	48 (21.92)	5 (2.28)	219 (15.62^†^)
Lumbar spinal stenosis with spondylolisthesis	Anterior fusion	0 (0)	0 (0)	1 (50)	0 (0)	1 (50)	0 (0)	2 (3.33)
	Posterior fusion	0 (0)	0 (0)	5 (31.25)	8 (50)	3 (18.75)	0 (0)	16 (26.67)
	Open discectomy	0 (0)	1 (3.03)	3 (9.09)	22 (66.67)	6 (18.18)	1 (3.03)	33 (55)
	Laminectomy	0 (0)	0 (0)	2 (22.22)	6 (66.67)	1 (11.11)	0 (0)	9 (15)
	Total	0 (0)	1 (1.67)	11 (18.33)	36 (60)	11 (18.33)	1 (1.67)	60 (11.34^†^)
Spondylolysis	Anterior fusion	0 (0)	0 (0)	0 (0)	1 (100)	0 (0)	0 (0)	1 (16.67)
	Open discectomy	0 (0)	0 (0)	2 (40)	3 (60)	0 (0)	0 (0)	5 (83.33)
	Total	0 (0)	0 (0)	2 (33.33)	4 (66.67)	0 (0)	0 (0)	6 (6.45^†^)

^†^The percentage of patients who underwent secondary surgery to the total patients for each diagnosis.

### Medical costs by diagnosis in each group

**Figure 2:**
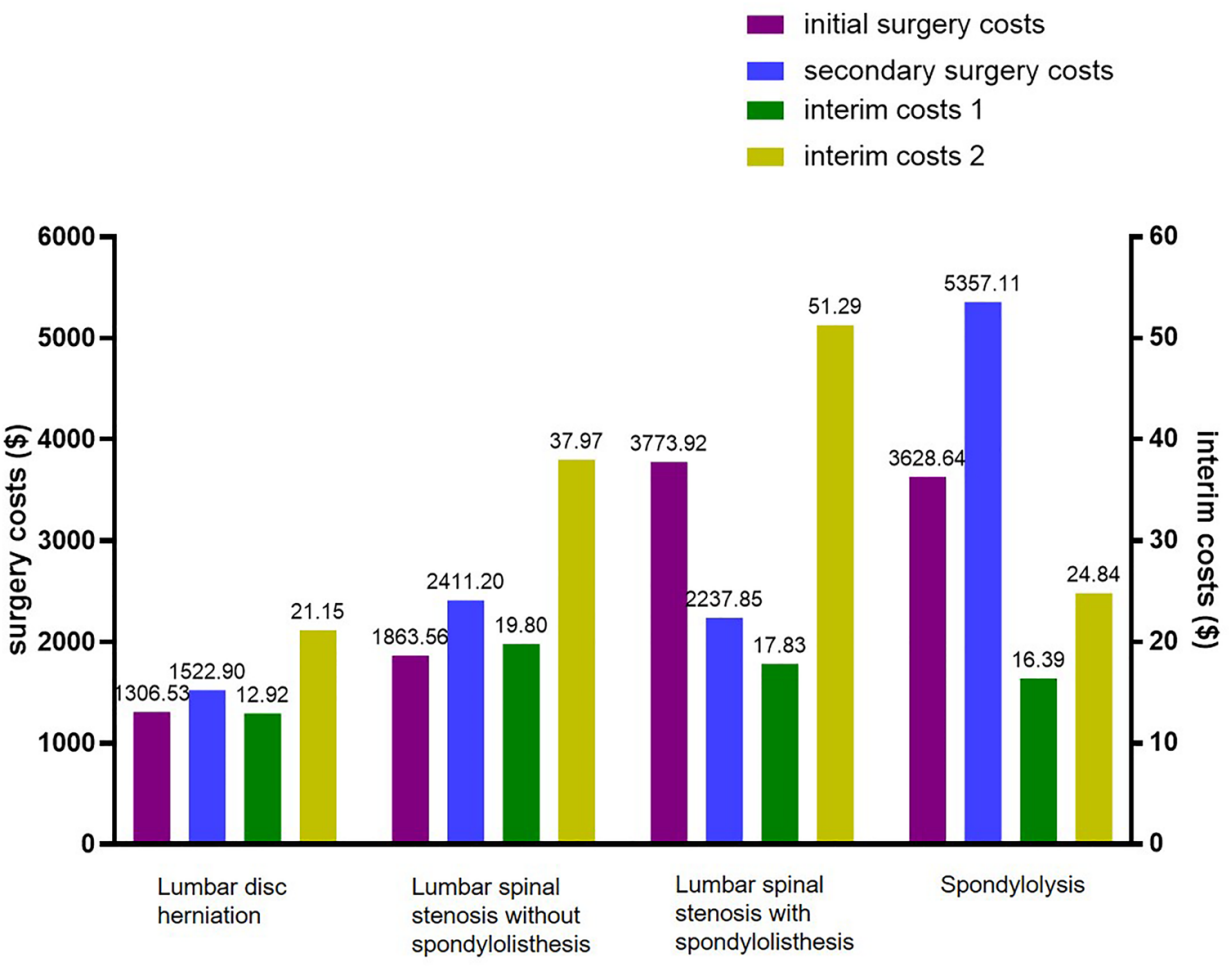
The comparison of interim costs and surgical costs based on diagnosis. Excluding patients of lumbar spinal stenosis with spondylolisthesis (LSS with SPL), secondary surgery costs of S-group were higher than initial surgery costs of NS-group. In all diagnosis, secondary surgery group (S-group) spent higher interim costs compared to non-secondary surgery group (NS-group).

The surgery costs and interim costs of the patients are presented in Table 4. The initial surgery costs were $1618.40 (range, 11.31–16,803.78), while the secondary surgery costs were $1829.59 (range, 9.89–19,988.60), which were higher than the initial surgery costs (P = 0.002). In LDH, LSS without SPL, and SP, the secondary surgery costs were higher than the initial surgery costs. However, the initial surgery costs were higher than the median secondary surgery costs in LSS with SPL. Before secondary surgery, the S-group incurred higher interim costs ($30.03; 1.86% of initial surgery costs) compared to the NS-group ($16.09; 0.99% of initial surgery costs). Higher interim costs before secondary surgery were observed in LDH (1.62% vs 0.99% of initial surgery costs), LSS without SPL (2.04% vs 1.06% of initial surgery costs), and LSS with SPL (1.36% vs 0.47% of initial surgery costs) in S-group than NS-group (P < 0.0001, < 0.0001, and < 0.0001, respectively). A comparison of initial, secondary, and interim costs for each diagnosis is presented in Fig. 2.

**Table 4:** The comparison of initial surgery costs, interim costs, and secondary surgery costs depending on each diagnosis.

Diagnosis	Initial surgery	Secondary surgery	Interim costs 1	Interim costs 2	P value^†^	P value^‡^
Lumbar disc herniation, median (range)	1306.53 (11.31–9552.88)	1522.90 (9.89–11,350.06)	12.92 (0.12–5218.59)	21.15 (0.19–10,944.19)		< 0.0001
Lumbar stenosis without spondylolisthesis, median (range)	1863.56 (314.46–16,803.78)	2411.20 (536.29–13,333.39)	19.80 (0.43–11,230.17)	37.97 (1.12–722.67)		< 0.0001
Lumbar stenosis with spondylolisthesis, median (range)	3773.92 (568.53–16,700.07)	2237.85 (540.00–19,988.60)	17.83 (1.24–807.21)	51.29 (0.23–327.96)		< 0.0001
Spondylolysis, median (range)	3628.64 (564.03–8406.16)	5357.11 (1089.78–12,417.99)	16.39 (1.76–169.88)	24.84 (1.28–138.43)		0.313
Total, median (range)	1618.40 (11.31–16,803.78)	1829.59 (9.89–19,988.60)	16.09 (0.12–11,230.17)	30.03 (0.19–10,944.19)	0.002	< 0.0001

All costs are monthly median medical costs ($), where $1 is equivalent to 1284.25 Korean won.

Interim costs 1, interim costs after initial surgery in non-secondary surgery group.

Interim costs 2, interim costs after initial surgery until secondary surgery in secondary surgery group.

^†^Comparison between initial surgery and secondary surgery.

^‡^Comparison between interim costs 1 and interim costs 2.

### The cutoff interim costs between S-group and NS-group

The cutoff interim costs for screening secondary surgery based on the surgical methods in each diagnosis of DLSD are presented in Table 5. For LDH, if interim costs after initial surgery were greater than $8.24 (0.63% of initial surgery costs), a secondary surgery could be predicted with sensitivity of 0.80 and specificity of 0.37. The cutoff value for predicting secondary surgery was $20.63 (1.58% of initial surgery costs; sensitivity of 1.00 and specificity of 0.51) for laminectomy as initial surgery and $16.83 (1.29% of initial surgery costs; sensitivity of 0.68 and specificity of 0.72) for endoscopic discectomy as initial surgery. The cutoff values were $25.16 (1.35% of initial surgery costs; sensitivity of 0.67 and specificity of 0.58) in LSS without SPL. For decompression as initial surgery, the cutoff value was $23.32 (1.25% of initial surgery; sensitivity of 0.71 and specificity of 0.56). The cutoff value was $28.42 (0.75% of initial surgery costs; sensitivity of 0.73 and specificity of 0.64) in LSS with SPL. The cutoff value for anterior fusion as initial surgery was $88.41 (2.34% of initial surgery costs; sensitivity of 1.00 and specificity of 0.96), and the cutoff value for posterior fusion as initial surgery was $20.50 (0.54% of initial surgery; sensitivity of 0.88 and specificity of 0.60). For decompression as initial surgery, the cutoff value was $28.69 (0.76% of initial surgery; sensitivity of 0.74 and specificity of 0.60).

**Table 5:** The cutoff interim costs for predicting secondary surgery by diagnosis and initial surgery methods.

Diagnosis	Initial surgery methods	Cutoff value ($)	AUC	Sensitivity	Specificity	P value
Lumbar disc herniation		8.24	0.61	0.80	0.37	< 0.0001
	Laminectomy	20.63	0.77	1.00	0.51	< 0.0001
	Endoscopic discectomy	16.83	0.73	0.68	0.72	0.0001
Lumbar spinal stenosis without spondylolisthesis		25.16	0.66	0.67	0.58	< 0.0001
	Anterior fusion	21.92	0.65	0.73	0.55	0.108
	Posterior fusion	8.59	0.61	1.00	0.22	0.144
	Decompression	23.32	0.67	0.71	0.56	< 0.0001
Lumbar spinal stenosis with spondylolisthesis		28.42	0.72	0.73	0.64	< 0.0001
	Anterior fusion	88.41	0.96	1.00	0.96	< 0.0001
	Posterior fusion	20.50	0.75	0.88	0.60	< 0.0001
	Decompression	28.69	0.69	0.74	0.60	< 0.0001
Spondylolysis		19.63	0.62	0.83	0.59	0.377
	Decompression	21.76	0.65	0.80	0.67	0.379

*AUC* area under the curve.

All costs are mean monthly medical costs ($), where $1 is equivalent to 1284.25 Korean won.

## Discussion

### Frequency and causes of secondary surgery in patients with degenerative lumbar spine disease

For LDH, the secondary surgery rate is reported to be 10% at 2 years, 15% at 5 years, and 20% at 10 years^11,32^. The most common cause of secondary surgery is known to be the recurrence of disc protrusion^16^. Factors such as age, gender, body mass index (BMI), smoking, and diabetes are known to contribute to the secondary surgery of LDH^33,34^. For LSS, secondary surgery is reported to occur at a rate of 11% to 18% between 8 and 10 years^13,35,36^. The main causes of secondary surgery are known to be the recurrence of stenosis due to disease progression or technical issues during surgery, accounting for about 50%^14,37^. Other causes include inadequate decompression, persistent pain, and complications resulting from the initial surgery^38–40^. Secondary surgery rates for degenerative SPL have been reported to range from 10 to 38% in previous literature^12,24,41^. Patients may undergo secondary surgery due to various reasons following the initial surgery, including facet joint hypertrophy, persistent pain, infection, and progression of degenerative changes^15,41,42^. The main complications that require secondary surgery in degenerative SPL are ASD and same segment disease (SSD). The risk factors associated with the occurrence of ASD and SSD are age, gender, BMI, facet tropism, disc height, and spinal instability^15,43^.

### The need to predict the occurrence of secondary surgery in degenerative lumbar spine disease

The prevalence of DLSD is increasing worldwide and it has placed a burden on healthcare budgets^44,45^. The growing burden of healthcare costs related to DLSD is a consequence of various factors, including an aging population, the increasing prevalence of the condition, the need for long-term management and treatment, advancements in medical technologies, the overall increase in use of medical resources, and increased number of secondary surgery^46–48^. In ROK, just like in other countries, the medical costs associated with DLSD are increasing^26^ and DLSD is placing a burden on the health insurance finances^26^. In this study, patients who underwent secondary surgery were found to incur significantly higher interim costs before secondary surgery compared to patients who did not undergo secondary surgery. In addition, the medical costs associated with secondary surgery were higher than the medical costs of the initial surgery. While many factors are known to be associated with the risk of secondary surgery for DLSD, there are no financial indicators for predicting secondary surgery^33,38,41,49^. In this study, interim costs after initial surgery showed promise in predicting the occurrence of secondary surgery in DLSD. Specifically, the study presented the cutoff interim costs that can predict secondary surgery based on the surgical methods for each diagnosis. Therefore, by tracking the post-surgical medical costs associated with DLSD, it may be possible to predict the occurrence of secondary surgery. Therefore, although it is an indirect indicator, the surrogate (interim costs) may indicate the number of patients having the possibility of secondary surgery. Screening secondary surgery in DLSD is a crucial factor in managing healthcare insurance budgets and can provide valuable information for the development of efficient healthcare policies.

### Limitations

Firstly, our pilot study used a sample cohort, which, while representing the national population, may not fully represent all cases of lumbar spine disease. Secondly, we hypothesized that higher medical costs could be linked to poor clinical outcomes. However, medical resource utilization varied among patients and doctors, and the study did not consider the impact of time on surgical outcomes^50^. Thirdly, the medical cost claims data lacked comprehensive clinical and imaging details. These limitations restricted our analysis of individual patient conditions, including the direct relationship between secondary and primary surgeries, and hindered our ability to fully assess patient-specific factors affecting surgical outcomes and subsequent healthcare costs. Fourthly, our analysis relied on medical cost data submitted to NHIS and did not consider factors like the patient's quality of life decline or losses due to unemployment. Additionally, non-insurance treatments were not included in the analysis.

## Conclusion

Among patients who underwent surgery for DLSD, those who underwent secondary surgery tend to have higher interim costs than those who did not undergo secondary surgery. Furthermore, secondary surgeries generally involve higher medical expenses than the initial surgery. Therefore, tracking the trend of medical costs increases in patients with DLSD who have undergone surgery can serve as an indicator for screening the need for secondary surgery.

## Data Availability

The datasets generated during and/or analyzed during the current study are available from the corresponding author on reasonable request.
